# Cocaine in Membranous Croup

**Published:** 1885-08

**Authors:** Sam’l. A. Greenwell

**Affiliations:** Cleburne, Tex.


### COCAINE IN MEMBRANOUS CROUP.

[FIRST CASE RECORDED.—ED.]

CLEBURNE, TEX., July 18, 1885.

F. E. DANIEL, M. D:

*Dear Sir:*—It is with great pleasure that I report a case of membranous croup, treated with muriate of cocaine with brilliant success. The cocaine was used in a two per cent solution, applied to the lower portion of the pharynx by means of hair pencil. Then a solution of the same applied to the larynx by means of a downward spray. I will forward to you a full report of the case for publication at some future time. I desire, now, to record the fact that I have used it as above stated, and in this I claim priority—without further comments.

I am Sir, Very Respectfully,
SAM'L A. GREENWELL, M. D.

